# High Baseline Neutrophil-to-Lymphocyte Ratio Could Serve as a Biomarker for Tumor Necrosis Factor-Alpha Blockers and Their Discontinuation in Patients with Ankylosing Spondylitis

**DOI:** 10.3390/ph16030379

**Published:** 2023-03-01

**Authors:** Dong-Hyuk Moon, Aran Kim, Byung-Wook Song, Yun-Kyung Kim, Geun-Tae Kim, Eun-Young Ahn, Min-Wook So, Seung-Geun Lee

**Affiliations:** 1 Division of Rheumatology, Department of Internal Medicine, Pusan National University Hospital, Pusan National University School of Medicine, Busan 49241, Republic of Korea; grayrheuma@naver.com (D.-H.M.); solees17@naver.com (A.K.); drsong@ynu.ac.kr (B.-W.S.); 2 Biomedical Research Institute, Pusan National University Hospital, Busan 49241, Republic of Korea; 3 Division of Rheumatology, Department of Internal Medicine, College of Medicine, Kosin University, Busan 49104, Republic of Korea; efmsungmo@hanmail.net (Y.-K.K.); gtah@hanmail.net (G.-T.K.); 4 Division of Rheumatology, Department of Internal Medicine, Pusan National University Yangsan Hospital, School of Medicine, Pusan National University, Yangsan 50612, Republic of Korea; ahnmolla@hanmail.net (E.-Y.A.); thalsdnrso@naver.com (M.-W.S.)

**Keywords:** ankylosing spondylitis (AS), tumor necrosis factor-alpha (TNF-α), treatment outcome, blood cells, biomarkers

## Abstract

Background: This study explores the association of neutrophil-to-lymphocyte (NLR), monocyte-to-lymphocyte (MLR), and platelet-to-lymphocyte (PLR) ratios with the 3-month treatment response and persistence of tumor necrosis factor-alpha (TNF-α) blockers in patients with ankylosing spondylitis (AS). Methods: This retrospective cohort study investigated 279 AS patients who were newly initiated on TNF-α blockers between April 2004 and October 2019 and 171 sex- and age-matched healthy controls. Response to TNF-α blockers was defined as a reduction in the Bath AS Disease Activity Index of ≥50% or 20 mm, and persistence referred to the time interval from the initiation to discontinuation of TNF-α blockers. Results: Patients with AS had significantly increased NLR, MLR, and PLR ratios as compared to controls. The frequency of non-response at 3 months was 3.7%, and TNF-α blockers’ discontinuation occurred in 113 (40.5%) patients during the follow-up period. A high baseline NLR but not high baseline MLR and PLR showed an independently significant association with a higher risk of non-response at 3 months (OR = 12.3, *p* = 0.025) and non-persistence with TNF-α blockers (HR = 1.66, *p* = 0.01). Conclusions: NLR may be a potential marker for predicting the clinical response and persistence of TNF-α blockers in AS patients.

## 1. Introduction

Ankylosing spondylitis (AS) is a chronic rheumatic disease that mainly affects the spine and sacroiliac joints, leading to progressive ankylosis of the axial skeleton. This condition also manifests as peripheral inflammatory arthritis, enthesitis, and extra-articular diseases such as psoriasis, uveitis, and inflammatory bowel diseases (IBD), which can reduce the quality of life and lead to disability and increased mortality [1]. The primary goals of AS treatment are symptom relief, improvement in physical function, and reduction in disease complications, including structural damage [2]. Both non-pharmacological and pharmacological therapies such as exercise and non-steroidal anti-inflammatory drugs (NSAIDs) are included in the treatment of AS. The advent of tumor necrosis factor-α (TNF-α) blockers has revolutionized the management of AS, owing to their remarkable efficacy in controlling the disease activity. TNF-α blockers are indicated for patients with AS who have an insufficient response to NSAIDs, and long-term use is recommended for these patients [2]. However, up to 40% of patients with AS showed limited efficacy to TNF-α blockers, and approximately half the patients with AS discontinued these drugs during the first five years of treatment due to various reasons [3,4]. Thus, the identification of predictors for the poor response and adherence of TNF-α blockers is important to optimize the management of AS. 

Recently, neutrophil-to-lymphocyte (NLR), monocyte-to-lymphocyte (MLR), and platelet-to-lymphocyte ratios (PLR) have gained attention as biomarkers that reflect the inflammatory status in rheumatic disorders, including AS [5,6], rheumatoid arthritis (RA) [7], and scleroderma [8] as well as malignancies [9] and cardiovascular diseases [10]. White blood cell and platelet counts can change in proportion to the inflammatory response, irrespective of the cause. In addition, neutrophils, monocytes, and lymphocytes are reported to be actively involved in the pathogenesis of various immune-mediated rheumatic diseases [11,12,13]. As neutrophil, monocyte, platelet, and lymphocyte counts are routinely measured in complete blood count tests in real clinical practice, NLR, MLR, and PLR are recognized as being easily accessible and inexpensive biomarkers. Previous studies have reported that these markers are associated with disease activity and radiographic severity in patients with AS [14,15,16,17,18], but only a few studies have evaluated their association with outcomes of the treatment with TNF-α blockers. Thus, in this study, we aimed to explore whether baseline NLR, MLR, and PLR can predict the 3-month treatment response and long-term persistence of TNF-α blockers in patients with AS.

## 2. Results

Table 1 summarizes the baseline data in patients with AS and healthy controls. Compared to healthy controls, the baseline NLR, MLR, and PLR in AS patients were significantly increased (NLR: 1.99 vs. 1.45; *p* < 0.001; MLR: 0.24 vs. 0.16; *p* < 0.001; PLR: 117.29 vs. 115.11; *p* = 0.036). The mean age was 34.5 years, and the majority of the patients were males (82.8%). The mean Bath AS Disease Activity Index (BASDAI) was 6.8, and adalimumab was the most frequently prescribed TNF-α blocker (66.3%), followed by etanercept (23.3%) and infliximab (10.4%). 

Supplementary Tables S1–S3 show comparisons of clinical data among patients with AS according to baseline NLR, MLR, and PLR levels, respectively. The high baseline NLR group had significantly increased C-reactive protein (CRP) levels and erythrocyte sedimentation rate (ESR), and a higher frequency of hip joint involvement as compared to the low baseline NLR group (Supplementary Table S1). The median CRP levels and ESR were significantly increased in patients in the high baseline MLR group as compared to those in the low baseline MLR group (Supplementary Table S2). As for PLR, a significantly higher CRP level and ESR as well as a higher frequency of females were observed in the high baseline group as compared to the low baseline group (Supplementary Table S3).

Table 2 describes the correlations of baseline NLR, MLR, and PLR with other clinical and laboratory findings. The correlation of baseline NLR, MLR, and PLR with ESR and CRP level was significantly positive. Additionally, baseline NLR and PLR but not MLR showed a significant positive correlation with baseline BASDAI (ρ = 0.132, *p* = 0.028 for baseline NLR; and ρ = 0.154, *p* = 0.01 for baseline PLR) and 3-month BASDAI (ρ = 0.137, *p* = 0.025 for baseline NLR; and ρ = 0.142, *p* = 0.02 for baseline PLR). 

Ten out of the 279 enrolled patients with AS discontinued TNF-α inhibitors therapy within the first 3 months of the index date, and the 3-month treatment response data were obtained only in the remaining 269 patients. Of these 269 patients, 10 (3.7%) did not achieve the 3-month treatment response to TNF-α blockers and were classified into the non-response group. The relationships of baseline NLR, MLR, and PLR with the 3-month treatment response, which were analyzed by logistic regression models, are summarized in Table 3. In the univariable logistic regression models, a high baseline NLR and the presence of IBD was significantly associated with non-response to 3-month TNF-α blockers, whereas baseline BASDAI was related with a lower possibility of non-response. High baseline MLR and PLR tended to be related to a higher risk of non-response, but the difference did not reach statistical significance. Multivariable logistic regression analyses found that a high baseline NLR was significantly associated with a higher risk of non-response to TNF-α blockers (odds ratio (OR) = 12.3, 95% confidence interval (CI) = 1.37–110.49, *p* = 0.025). After adjusting for confounders, high baseline MLR and PLR tended to increase the possibility of non-response, but this association did not show statistical significance (OR = 4.8, 95% CI = 0.94–24.53, *p* = 0.059 for high baseline MLR, and OR = 4.63, 95% CI = 0.89–24.18, *p* = 0.069 for high baseline PLR). 

**Figure 1:**
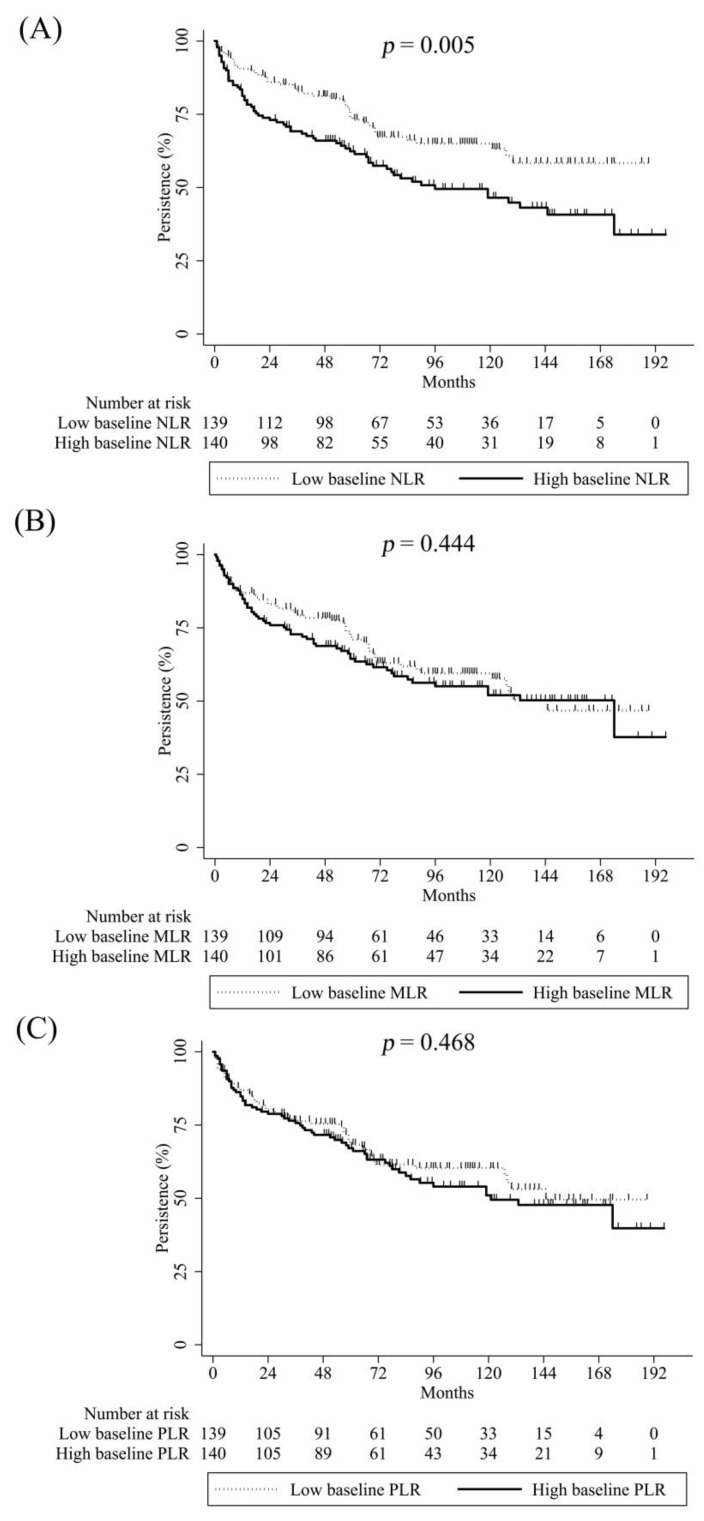
Comparisons of drug persistence of tumor necrosis factor-alpha blockers in patients with ankylosing spondylitis according to the baseline neutrophil-to-lymphocyte (**A**), monocyte-to-lymphocyte (**B**), and platelet-to-lymphocyte ratios (**C**).

Discontinuation of TNF-α blockers occurred in 113 (40.5%) patients during the follow-up period. The Kaplan–Meier curve for drug persistence in all the study patients is shown in Supplementary Figure S1. The one-, two-, and five-year persistence rates of the TNF-α blockers were 85.6, 79.5, and 67.7%, respectively. Poor health literacy (*n* = 61, 54%) was the most common cause of TNF-α blockers’ discontinuation, followed by lack of efficacy (LOE) (*n* = 29, 25.7%) and adverse events (AE) (*n* = 23, 20.4%). As shown in Figure 1, AS patients with low baseline NLR showed better drug persistence compared to those with high baseline NLR. However, there was no significant difference in drug persistence according to the baseline MLR and PLR levels (Figure 1). Factors associated with non-persistence of TNF-α blockers are shown in Table 4. In the univariate Cox proportional hazard regression models, high baseline NLR, psoriasis, and hip involvement were significantly associated with a higher risk of discontinuation of TNF-α blockers. Female sex tended to be associated with a higher risk of non-persistence, and the presence of uveitis showed the trend of a lower possibility of TNF-α blockers’ withdrawal. Meanwhile, high baseline MLR and PLR did not show a significant association with non-persistence of TNF-α blockers. Multivariable Cox proportional hazard regression models found that high baseline NLR had a significant hazard ratio of 1.66 (95% CI = 1.13–2.44, *p* = 0.01) for TNF-α blockers’ discontinuation. Additionally, female sex and hip involvement were significantly related to non-persistence for TNF-α blockers, after the adjustment of potential confounders.

## 3. Discussion

The current study analyzed the role of baseline NLR, MLR, and PLR for the prediction of the clinical response and continuation of anti-TNF-α drugs in patients with AS. These three markers were significantly higher in patients with AS as compared to healthy controls, and were positively correlated with serum CRP and ESR levels. Additionally, NLR and PLR in patients with AS had a weak positive correlation with disease activity, as assessed by the BASDAI. After the adjustment of potential confounders, a high baseline NLR was significantly linked with a higher risk of a poor treatment response and discontinuation of TNF-α blockers in patients with AS, but this relationship was not found in those with high baseline MLR and PLR, respectively. The findings of our study suggest that NLR not only reflects disease activity but can also predict the clinical response and long-term adherence of TNF-α blockers in patients with AS. 

Similar to our results, two recent meta-analyses demonstrated that NLR levels in patients with AS were significantly higher than those in healthy participants [5,6]. Our data also showed that, similar to NLR, PLR and MLR were also significantly increased in AS as compared to controls. Although conflicting results have also been reported in the literature [19,20], previous studies have found that NLR and PLR in AS patients display a positive correlation with disease activity indices such as BASDAI [14,15,16,17] and inflammatory indicators such as CRP and ESR [21], which is in line with our current findings. Liang et al. showed that PLR could be a useful indicator for AS diagnosis, and that it is associated with the radiographic severity of AS [18]. Thus, it is suggested that NLR and PLR were indicative for the underlying inflammatory burden in AS. ESR and CRP have been used to assess the diagnosis and disease activity of AS, but their sensitivities and specificities are not always satisfactory [22,23]. Not all patients with active AS have elevated ESR and CRP levels [16]. Therefore, for assessing inflammatory status such as the disease activity of AS, NLR and PLR may be at least as good as ESR and CRP, although further studies are needed to validate this hypothesis. In addition, the high baseline NLR group had an increased frequency of hip joint involvement than the low baseline NLR group (Supplementary Table S1), suggesting that NLR could also reflect the severity of AS. Although MLR was positively correlated with ESR and CRP levels, it did not show a significant association with BASDAI in our data. Similarly, Huang et al. reported that MLR had a close relationship with ESR, CRP, finger-to-floor distance, and modified Schober tests [21]. However, in contrast to NLR and PLR, there is a lack of evidence to support a significant association between MLR and disease activity in AS according to our literature review.

Drug persistence is interpreted as a composite clinical measure of efficacy and safety in real-world situations. One of the major results of this study was that a high baseline NLR was a significantly predictive factor for the risk of TNF-α blockers’ non-persistence in patients with AS. Previous studies have reported that in patients with AS, elevated levels of CRP and ESR and increased disease activity are significantly associated with the risk of the discontinuation of TNF-α inhibitors [24,25,26]. These findings suggest that a higher inflammatory burden in patients with AS may contribute to an increased risk of the non-persistence of TNF-α blockers. As mentioned above, NLR is recognized as a surrogate marker that is reflective of the systemic inflammatory response, which can explain its association with non-persistence to anti-TNF-α agents in our data. We observed that hip joint involvement, which indicates more severe disease, was related to a higher risk of discontinuing TNF-α inhibitors, which was in line with a study by Jeong et al. [25] Additionally, complete spinal ankylosis and a higher grade of sacroiliitis were related to a higher risk of the non-persistence of TNF-α blockers in previous reports [27,28]. Overall, it is reasonable to consider that patients with AS with higher severity and disease activity are more likely to discontinue anti-TNF-α agents.

We also observed that a higher baseline NLR was significantly predictive for a 3-month treatment response of TNF-α blockers in patients with AS. Multiple studies have reported that lower baseline disease activity is a significant predictor for remission in patients with AS after TNF-α inhibitors’ treatment [29,30,31], indicating that a higher baseline inflammatory status can be linked with a poor treatment response. Numerous studies, including our study, support the significant relationship between NLR and disease activity in patients with AS [14,15,16,17], whereby NLR can also be linked with the treatment response to TNF-α blockers. Additionally, Coskun et al. reported that NLR in patients with AS significantly decreased after treatment for 3 months with anti-TNF-α drugs [17]. Similar to AS, increased baseline NLR levels were found to determine the 12-week treatment response to TNF-α blockers in RA patients in a study by Lee et al. [7] Taken together, we believe that NLR may serve as a parameter for monitoring and predicting the therapeutic response to TNF-α blockers in patients with inflammatory rheumatic diseases, including AS. 

Unlike NLR, baseline MLR and PLR in patients with AS did not show a significant association with the treatment response and continuation of TNF-α inhibitors in our study, although they significantly correlated with ESR and CRP. Thus, neutrophils may be more closely related to the action of TNF-α in the pathogenesis of AS than monocytes and platelets. However, this was somewhat unexpected because macrophages/monocytes, but not neutrophils, are a major source of TNF-α [32]. Interleukin (IL))-17, another important pro-inflammatory cytokine in the pathogenetic process of AS, mainly originates in the T helper 17 cells; however, neutrophils can also produce this cytokine [33]. Appel et al. reported that neutrophils are the major source of the local synthesis of IL-17 in the zygapophyseal joints of patients with AS [34]. The interplay between TNF-α and IL-17 in AS pathogenesis is largely unknown, but it is likely that these two cytokines are closely related. Xueyi et al. investigated the changes in the serum levels of pro-inflammatory cytokines in patients with AS during TNF-α inhibitor therapy, and reported that serum concentrations of IL-17 were significantly reduced in subjects with a good response to this therapy [35], which supports this notion. Taken together, these findings suggest that neutrophils may be linked to the role of TNF-α in the inflammatory process of AS via IL-17. In previous studies of patients with AS, monocytes exhibited higher pro-inflammatory properties, and showed more pronounced phagocytic activity as compared to controls [36]. Although platelets can be involved in the inflammatory process by secreting various cytokines such as IL-6 and transforming growth factor-β, the role of platelets in the pathophysiology of AS has not been well studied. 

This study has a number of limitations that need to be addressed. First, because this was a retrospective cohort study conducted in a single center, all potential confounding factors could not be fully adjusted. Second, the causal relationship of NLR with clinical response and persistence to TNF-α inhibitors could not be fully established because NLR was measured only once in our study. In addition, we only assessed the 3-months clinical response of TNF-α inhibitors, which can be biased by reverse causation or unknown confounding factors. Thus, longitudinal changes in the NLR in patients with AS need to be investigated to determine the causal relationship between NLR and treatment outcome. Third, the small number of AS patients with a poor treatment response to anti-TNF-α agents may have led to insufficient statistical power. 

## 4. Materials and Methods

### 4.1. Study Designs and Participants

This is a retrospective cohort study using electronic medical records from a tertiary referral center in South Korea. In the present study, we analyzed data of 279 patients with AS and compared them with 171 sex- and age (±2 years)-matched healthy controls. For age matching, the patients with AS and the healthy controls were matched by year of birth. If no control was found, this age-matching criterion was expanded stepwise, in age decrements or increments of 1 year to a maximum of 2 years. If there was no eligible healthy subject that could be matched to an AS patient within 2 years of age, we only evaluated the data of healthy controls. For sex matching, we matched the male-to-female ratio between AS patient and control groups. These patients with AS had been newly prescribed with TNF-α blockers as the first-line biological treatment due to poor response to NSAIDs between April 2004 and October 2019 by experienced rheumatologists. All patients with AS met the modified New York Criteria [37] and were followed-up until December 2021. During the follow-up period, adalimumab, etanercept, and infliximab were available at our center, and the index date referred to the start date of anti-TNF-α agents. In South Korea, nearly the entire population (98%) is enrolled in the Korean National Health Insurance Service (NHIS) program, and anti-TNF-α agents are eligible for reimbursement by the NHIS program in the following conditions: when patients with AS have a Bath AS Disease Activity Index (BASDAI) > 4 with inadequate response to two or more types of NSAIDs or disease-modifying antirheumatic drugs (DMARDs) or when NSAIDs or DMARDs are discontinued due to their side effects. Exclusion criteria in our study were as follows: patients with AS (1) <18 years; (2) those treated with TNF-α blockers or IL-17 inhibitors within 6 months prior to the index date; (3) those who received TNF-α blockers for the management of rheumatic or autoimmune diseases other than AS, such as RA; (4) those with concomitant active infection; and (5) those with underlying hemato-oncological diseases. Data were obtained for healthy controls who received comprehensive health checkups at the health promotion department of the same center and did not have any rheumatic disorders including AS, active infection, and hemato-oncological diseases. The present study was approved by the Institutional Review Board of Pusan National University Hospital, and requirement for informed consent was waived because this was a retrospective study (IRB No. 2207-003-016, approval date 12 July 2022).

### 4.2. Covariates

Absolute counts of neutrophils, monocytes, lymphocytes, platelets, and CRP were measured at the index date (±14 days) for the participants in this study. NLR, MLR, and PLR were calculated as follows: absolute count of neutrophil ÷ absoluted count of lymphocyte; absolute count of monocyte ÷ absolute count of lymphoycte; absolute count of platelet ÷ absolute count of lymphocyte, respectively. 

In patients with AS, data regarding ESR, disease duration, BASDAI, type of TNF-α blockers, HLA-B27, presence of previous and/or current uveitis, psoriasis, peripheral arthritis, hip joint involvement, IBD, and concomitant medications were obtained. Disease duration referred to the time period between the AS diagnosis date and the index date. Uveitis referred to an episode of ophthalmic diagnosis by an ophthalmologist, and psoriasis was defined as being diagnosed by a dermatologist. Peripheral arthritis referred to the presence of one or more swollen joint in the past or present, excluding the hip joints [38]. Hip joint involvement referred to localized pain, joint motion limitation, or lameness together with radiographic findings of joint space narrowing, bone ankylosis, subchondral erosion, sclerosis, subluxation, and other deformities [39]. IBD was defined as either a diagnosis by a gastroenterologist or by conventional colonoscopy and pathologic criteria [40]. Concomitant medications included NSAIDs, methotrexate, sulfasalazine, and glucocorticoids.

### 4.3. Study Outcomes

Treatment response was assessed after the use of TNF-α blockers for 3 months. Furthermore, a decrease in BASDAI of ≥50% or 20 mm compared with baseline BASDAI was considered as having a clinical response [41]. Because achievement of 3-months response of TNF-α blockers is known to be associated with long-term clinical outcome of patients with AS, we selected 3-months time point as one of the main study outcomes. In Korea, if a 3-month treatment response is achieved, an additional 6 months of TNF-α blockers use can be reimbursed by the NHIS. Hence, in the present study, BASDAI was evaluated every 6 months, and if the 3-month treatment response was maintained, TNF-α blockers’ administration was reimbursed for an additional 6 months.

Drug persistence was defined as the time duration between the index date and the date of discontinuation of TNF-α blockers, and was expressed in months. If the index TNF-α blockers were switched to other TNF-α blockers or IL-17 inhibitors, or the index TNF-α blockers were restarted >90 days (permissible gap) after the last prescription, it was considered to be non-persistent to TNF-α blockers. Literature review indicated that 90 days permissible gap has been widely used for studying the long-term adherence of TNF-α blockers for the treatment of rheumatic diseases [7,42,43,44,45]. Causes of discontinuation of anti-TNF-α agents were classified as LOE, AE, and poor health literacy based on medical chart review. Lack of efficacy indicates failure to respond to anti-TNF-α agents or worsening of disease activity. Poor health literacy referred to the discontinuation of TNF-α inhibitors, owing to unawareness of the significance of regular use of these medications in the management of AS [46].

### 4.4. Statistical Methods

Mean ± standard deviation (SD), median (interquartile range), or number (percent) were used to express continuous and categorical variables. Student’s t-test, Mann–Whitney U test, chi-square test, or Fisher’s exact test were applied for group comparisons. Correlations between continuous variables with or without normal distributions were estimated using the Spearman’s correlation analysis. Drug persistence of TNF-α blockers were estimated by Kaplan–Meier curves and compared with log-rank test. The relationship of baseline NLR, MLR, and PLR with 3-month treatment response to TNF-α blockers was assessed using multivariable logistic regression models adjusted for variables with *p* < 0.1 in the univariable models. To assess whether baseline NLR, MLR, and PLR were independently associated with the risk of TNF-α blockers’ discontinuation, we used multivariable Cox proportional hazard regression models that included variables with *p* < 0.1 in the univariable models. Baseline NLR, MLR, and PLR were dichotomized using a median split (high versus low groups) and included in both logistic regression and Cox proportional hazard regression models. Because the baseline NLR, MLR, and PLR were highly correlated with each other, these variables were separately analyzed in the multivariable logistic and regression models to prevent the possibility of multicollinearity. We regarded *p* < 0.05 as statistical significance, and used STATA version 15.0 of Windows software (StataCorp LP, College Station, TX, USA) for statistical analyses.

## 5. Conclusions

In summary, the present study suggests that NLR serves as a useful marker for detecting a 3-months clinical response and persistence of TNF-α blockers in patients with AS. Because NLR is inexpensive, easily available, and a routinely measured parameter in real-world clinical practice, we believe that it can be a valuable and useful biomarker in the assessment of clinical outcomes in patients with AS. However, the baseline MLR and PLR did not show these associations. However, due to the limitations of this study, further longitudinal studies are warranted to confirm our results.

## Figures and Tables

**Table 1:** Baseline clinical characteristics in patients with ankylosing spondylitis and healthy controls.

	Patients with AS (*n* = 279)	Healthy Controls (*n* = 171)	*p*-Value
Age, years, mean ± SD	34.5 ± 11.1	34.9 ± 6.4	0.655
Female, *n* (%)	48 (17.2)	30 (17.5)	0.926
NLR, median (IQR)	1.99 (1.36–2.72)	1.45 (1.17–1.83)	<0.001
MLR, median (IQR)	0.24 (0.18–0.33)	0.16 (0.13–0.2)	<0.001
PLR, median (IQR)	117.29 (94.91–158.91)	115.11 (90.94–137.37)	0.036
CRP, mg/dL, median (IQR)	0.93 (0.24–2.57)	0.03 (0.02–0.05)	<0.001
ESR, mm/h, median (IQR)	25.5 (9.3–52.8)		
Disease duration, months, median (IQR)	8 (4–43)		
BASDAI, mean ± SD	6.8 ± 1.4		
Ant–TNF–α agents			
Adalimumab, *n* (%)	185 (66.3)		
Etanercept, *n* (%)	65 (23.3)		
Infliximab, *n* (%)	29 (10.4)		
HLA–B27, *n* (%)	221 (88)		
Peripheral arthritis, *n* (%)	125 (44.8)		
Hip joint involvement, n (%)	89 (31.9)		
Uveitis, *n* (%)	60 (21.5)		
Psoriasis, *n* (%)	11 (3.9)		
IBD, *n* (%)	7 (2.5)		
Concomitant medications			
NSAIDs, *n* (%)	200 (71.7)		
Methotrexate, *n* (%)	60 (21.5)		
Sulfasalazine, *n* (%)	88 (31.5)		
Glucocorticoids s, *n* (%)	101 (36.2)		

AS: ankylosing spondylitis, SD: standard deviation, NLR: neutrophil-to-lymphocyte ratio, IQR: interquartile range, MLR: monocyte-to-lymphocyte ratio, PLR: platelet-to-lymphocyte ratio, CRP: C-reactive protein, ESR: erythrocyte sedimentation rate, BASDAI: Bath Ankylosing Spondylitis Disease Activity Index, TNF-α: tumor necrosis factor-α, HLA: human leukocyte antigen, IBD: inflammatory bowel disease, NSAIDs: non-steroidal anti-inflammatory drugs.

**Table 2:** Correlations between clinical and laboratory markers in patients with ankylosing spondylitis.

	1	2	3	4	5	6	7
1. Baseline NLR	-	-	-	-	-	-	-
2. Baseline MLR	0.712 **	-	-	-	-	-	-
3. Baseline PLR	0.703 **	0.613 **	-	-	-	-	-
4. Baseline ESR	0.33 **	0.288 **	0.364 **	-	-	-	-
5. Baseline CRP	0.358 **	0.358 **	0.346 **	0.766 **	-	-	-
6. Baseline BASDAI	0.132 *	0.041	0.154 **	0.172 **	0.091	-	-
7. 3-month BASDAI	0.137 *	0.119	0.142 *	0.098	0.033	0.439 **	-
8. 3-month changes in BASDAI	−0.004	−0.069	0.027	0.09	0.035	0.601 **	−0.364 **

* *p* < 0.05, ** *p* < 0.001, NLR: neutrophil-to-lymphocyte ratio, MLR: monocyte-to-lymphocyte ratio, PLR: platelet-to-lymphocyte ratio, ESR: erythrocyte sedimentation rate, CRP: C-reactive protein, BASDAI: Bath Ankylosing Spondylitis Disease Activity Index.

**Table 3:** Associated factors for non-response to tumor necrosis factor-alpha inhibitors in patients with ankylosing spondylitis.

	Crude OR (95% CI)	*p*-Value	Adjusted OR * (95% CI)	*p*-Value	Adjusted OR * (95% CI)	*p*-Value	Adjusted OR * (95% CI)	*p*-Value
High baseline NLR	9.65 (1.21–77.25)	0.033	12.3 (1.37–110.49)	0.025				
High baseline MLR	4.16 (0.87–19.96)	0.075			4.8 (0.94–24.53)	0.059		
High baseline PLR	4.09 (0.85–19.65)	0.078					4.63 (0.89–24.18)	0.069
Disease duration, month	1.01 (0.99–1.02)	0.064	1.01 (0.99–1.03)	0.069	–	–	1.01 (0.99–1.02)	0.088
BASDAI	0.59 (0.35–0.97)	0.038	0.59 (0.34–1.02)	0.059	0.57 (0.33–0.97)	0.038	0.61 (0.36–1.02)	0.059
IBD	12.7 (2.13–75.65)	0.005	10.24 (1.45–72.37)	0.02	9.22 (1.32–64.61)	0.025	9.95 (1.45–68.51)	0.02
Age	0.99 (0.94–1.05)	0.856						
Female	1.16 (0.24–5.63)	0.856						
CRP, mg/dL	0.96 (0.74–1.24)	0.731						
Uveitis	1.52 (0.38–6.06)	0.554						
Peripheral arthritis	3.06 (0.78–12.1)	0.111						
Hip involvement	1.44 (0.4–5.24)	0.581						
NSAIDs	0.6 (0.17–2.19)	0.439						
Methotrexate	0.93 (0.19–4.5)	0.925						
Sulfasalazine	2.28 (0.64–8.09)	0.203						
Glucocorticoids	0.43 (0.09–2.07)	0.294						

* Adjusted OR: adjusted for BASDAI, disease duration, and IBD. OR: odds ratio, CI: confidence interval, NLR: neutrophil-to-lymphocyte ratio, MLR: monocyte-to-lymphocyte ratio, PLR: platelet-to-lymphocyte ratio, BASDAI: Bath Ankylosing Spondylitis Disease Activity Index, IBD: inflammatory bowel disease, CRP: C-reactive protein, NSAIDs: non-steroidal anti-inflammatory drugs.

**Table 4:** Associated factors for drug persistence to tumor necrosis factor-alpha inhibitors in patients with ankylosing spondylitis.

	Crude HR (95% CI)	*p*-Value	Adjusted HR * (95% CI)	*p*-Value
High baseline NLR	1.7 (1.17–2.48)	0.006	1.66 (1.13–2.44)	0.01
High baseline MLR	1.16 (0.8–1.67)	0.446		
High baseline PLR	1.14 (0.79–1.66)	0.47		
Female	1.46 (0.95–2.26)	0.085	1.56 (1.01–2.41)	0.048
Uveitis	0.66 (0.4–1.08)	0.094	0.63 (0.39–1.03)	0.067
Psoriasis	2.32 (1.07–4.99)	0.032	2.11 (0.98–4.57)	0.057
Hip involvement	1.6 (1.1–2.32)	0.014	1.48 (1.01–2.16)	0.042
TNF-α inhibitors Adalimumab Etanercept Infliximab (ref.)	0.89 (0.58–1.37) 0.8 (0.41–1.56)	0.599 0.513		
Sulfasalazine	1.47 (1.01–2.14)	0.049	-	-
Age	0.99 (0.98–1.01)	0.408		
Disease duration, month	1 (0.99–1)	0.371		
BASDAI	0.91 (0.79–1.04)	0.155		
CRP, mg/dL	1.03 (0.98–1.08)	0.289		
Peripheral arthritis	0.97 (0.67–1.4)	0.856		
IBD	0.97 (0.31–3.05)	0.956		
NSAIDs	1.39 (0.91–2.12)	0.124		
Methotrexate	0.97 (0.62–1.51)	0.882		
Glucocorticoids	1.33 (0.91–1.93)	0.136		

* Adjusted HR: adjusted for NLR, female, uveitis, psoriasis, hip involvement, and SSZ. HR: hazard ratio, CI: confidence interval, NLR: neutrophil-to-lymphocyte ratio, MLR: monocyte-to-lymphocyte ratio, PLR: platelet-to-lymphocyte ratio, TNF-α: tumor necrosis factor-α, BASDAI: Bath Ankylosing Spondylitis Disease Activity Index, CRP: C-reactive protein, IBD: inflammatory bowel disease, NSAIDs: non-steroidal anti-inflammatory drugs.

## Data Availability

Data are contained within the article and the Supplementary Materials.

## Supplementary Materials

The following supporting information can be downloaded at: https://www.mdpi.com/1424-8247/16/3/379/s1, Figure S1: Drug persistence of tumor necrosis factor-alpha blockers in all the study patients; Table S1: Comparisons of clinical and laboratory characteristics in patients with ankylosing spondylitis according to the high and low baseline neutrophil-to-lymphocyte ratio; Table S2: Comparisons of clinical and laboratory characteristics in patients with ankylosing spondylitis according to the high and low baseline monocyte-to-lymphocyte ratio; Table S3: Comparisons of clinical and laboratory characteristics in patients with ankylosing spondylitis according to the high and low baseline platelet-to-lymphocyte ratio.
