# Supplementary material for: High Baseline Neutrophil-to-Lymphocyte Ratio Could Serve as a Biomarker for Tumor Necrosis Factor-Alpha Blockers and Their Discontinuation in Patients with Ankylosing Spondylitis
Source: Pharmaceuticals (Basel). 2023 Mar 1;16(3):379. doi: 10.3390/ph16030379 (PMC10055887; doi:10.3390/ph16030379)
Supplement: Supplementary file 1 [file pharmaceuticals-16-00379-s001.zip › Supplmentary materials/Supplementary Table 1.pdf]

Supplementary Table S1. Comparisons of clinical and laboratory characteristics in patients with ankylosing spondylitis according to the high and low baseline neutrophil-to-lymphocyte ratio.

	Low baseline NLR (n = 139)	High baseline NLR (n = 140)	p value
Age, years, mean ± SD	34.3 ± 10.9	34.7 ± 11.4	0.762
Female, n (%)	28 (20.1)	20 (14.3)	0.208
CRP, mg/dL, median (IQR)	0.44 (0.08–1.38)	1.44 (0.57–3.65)	<0.001
ESR, mm/hr, median (IQR)	5 (15-33)	35 (20-62.5)	<0.001
Disease duration, months, median (IQR)	8 (4-64)	4 (8-34.8)	0.281
BASDAI, mean ± SD	6.8 ± 1.3	6.8 ± 1.4	0.684
TNF-α inhibitors			0.061
Adalimumab, n (%)	101 (72.7)	84 (60)	
Etanercept, n (%)	28 (20.1)	37 (26.4)	
Infliximab, n (%)	10 (7.2)	19 (13.6)	
HLA-B27, n (%)	115 (88.5)	106 (87.6)	0.834
Peripheral arthritis, n (%)	58 (41.7)	57 (47.9)	0.336
Hip joint involvement, n (%)	34 (24.5)	55 (39.3)	0.01
Uveitis, n (%)	28 (20.1)	32 (22.9)	0.662
Psoriasis, n (%)	5 (3.6)	6 (4.3)	0.768
IBD, n (%)	2 (1.4)	5 (3.6)	0.447

SD: standard deviation, NLR, neutrophil-to-lymphocyte ratio, CRP: C-reactive protein, IQR: inter-quartile range, ESR: erythrocyte sedimentation rate, BASDAI: Bath Ankylosing Spondylitis Disease Activity Index, TNF-α: tumor necrosis factor-α, HLA: human leukocyte antigen, IBD: inflammatory bowel disease.
