# Supplementary material for: High Baseline Neutrophil-to-Lymphocyte Ratio Could Serve as a Biomarker for Tumor Necrosis Factor-Alpha Blockers and Their Discontinuation in Patients with Ankylosing Spondylitis
Source: Pharmaceuticals (Basel). 2023 Mar 1;16(3):379. doi: 10.3390/ph16030379 (PMC10055887; doi:10.3390/ph16030379)
Supplement: Supplementary file 1 [file pharmaceuticals-16-00379-s001.zip › Supplmentary materials/Supplementary Table 2.pdf]

Supplementary Table S2. Comparisons of clinical and laboratory characteristics in patients with ankylosing spondylitis according to the high and low baseline monocyte-to-lymphocyte ratio.

	Low baseline MLR (n = 139)	High baseline MLR (n = 140)	p value
Age, years, mean ± SD	33.8 ± 10.8	35.2 ± 11.4	0.289
Female, n (%)	27 (19.4)	21 (15)	0.345
CRP, mg/dL, median (IQR)	0.47 (0.1–1.41)	1.4 (0.51–3.65)	<0.001
ESR, mm/hr, median (IQR)	16 (5.8–37.3)	32.5 (17–59.5)	<0.001
Disease duration, months, median (IQR)	8 (4–48)	7.5 (4–41.3)	0.517
BASDAI, mean ± SD	6.7 ± 1.4	6.8 ± 1.3	0.554
TNF-α inhibitors			0.312
Adalimumab, n (%)	97 (69.8)	88 (62.9)	
Etanercept, n (%)	27 (19.4)	38 (27.1)	
Infliximab, n (%)	15 (10.8)	14 (10)	
HLA-B27, n (%)	116 (88.5)	105 (87.5)	0.798
Peripheral arthritis, n (%)	58 (41.7)	67 (47.9)	0.336
Hip joint involvement, n (%)	40 (28.8)	49 (35)	0.305
Uveitis, n (%)	31 (22.3)	29 (20.7)	0.772
Psoriasis, n (%)	7 (5)	4 (2.9)	0.35
IBD, n (%)	3 (2.2)	4 (2.9)	1

SD: standard deviation, MLR, monocyte-to-lymphocyte ratio, CRP: C-reactive protein, IQR: inter-quartile range, ESR: erythrocyte sedimentation rate, BASDAI: Bath Ankylosing Spondylitis Disease Activity Index, TNF-α: tumor necrosis factor-α, HLA: human leukocyte antigen, IBD: inflammatory bowel disease.
