# Supplementary material for: High Baseline Neutrophil-to-Lymphocyte Ratio Could Serve as a Biomarker for Tumor Necrosis Factor-Alpha Blockers and Their Discontinuation in Patients with Ankylosing Spondylitis
Source: Pharmaceuticals (Basel). 2023 Mar 1;16(3):379. doi: 10.3390/ph16030379 (PMC10055887; doi:10.3390/ph16030379)
Supplement: Supplementary file 1 [file pharmaceuticals-16-00379-s001.zip › Supplmentary materials/Supplementary Table 3.pdf]

Supplementary Table S3. Comparisons of clinical and laboratory characteristics in patients with ankylosing spondylitis according to the high and low baseline platelet-to-lymphocyte ratio.

	Low baseline PLR (n = 139)	High baseline PLR (n = 140)	p value
Age, years, mean ± SD	35.7 ± 11.3	33.3 ± 10.8	0.073
Female, n (%)	17 (12.2)	31 (22.1)	0.038
CRP, mg/dL, median (IQR)	0.62 (0.13–1.56)	1.37 (0.42–3.55)	<0.001
ESR, mm/hr, median (IQR)	16.5 (6–32.3)	35.5 (17–62.3)	<0.001
Disease duration, months, median (IQR)	6 (4–39)	12 (4–43)	0.469
BASDAI, mean ± SD	6.7 ± 1.3	6.9 ± 1.4	0.216
TNF-α inhibitors			0.035
Adalimumab, n (%)	102 (73.4)	83 (59.3)	
Etanercept, n (%)	24 (17.3)	41 (29.3)	
Infliximab, n (%)	13 (9.4)	16 (11.4)	
HLA-B27, n (%)	112 (87.5)	109 (88.6)	0.847
Peripheral arthritis, n (%)	55 (39.6)	70 (50)	0.092
Hip joint involvement, n (%)	39 (28.1)	50 (35.7)	0.199
Uveitis, n (%)	32 (23)	28 (20)	0.539
Psoriasis, n (%)	6 (4.3)	5 (3.6)	0.769
IBD, n (%)	2 (1.4)	5 (3.6)	0.447

SD: standard deviation, PLR, platelet-to-lymphocyte ratio, CRP: C-reactive protein, IQR: inter-quartile range, ESR: erythrocyte sedimentation rate, BASDAI: Bath Ankylosing Spondylitis Disease Activity Index, TNF-α: tumor necrosis factor-α, HLA: human leukocyte antigen, IBD: inflammatory bowel disease.
