# Supplementary figures and images for: High Baseline Neutrophil-to-Lymphocyte Ratio Could Serve as a Biomarker for Tumor Necrosis Factor-Alpha Blockers and Their Discontinuation in Patients with Ankylosing Spondylitis
Source: Pharmaceuticals (Basel). 2023 Mar 1;16(3):379. doi: 10.3390/ph16030379 (PMC10055887; doi:10.3390/ph16030379)

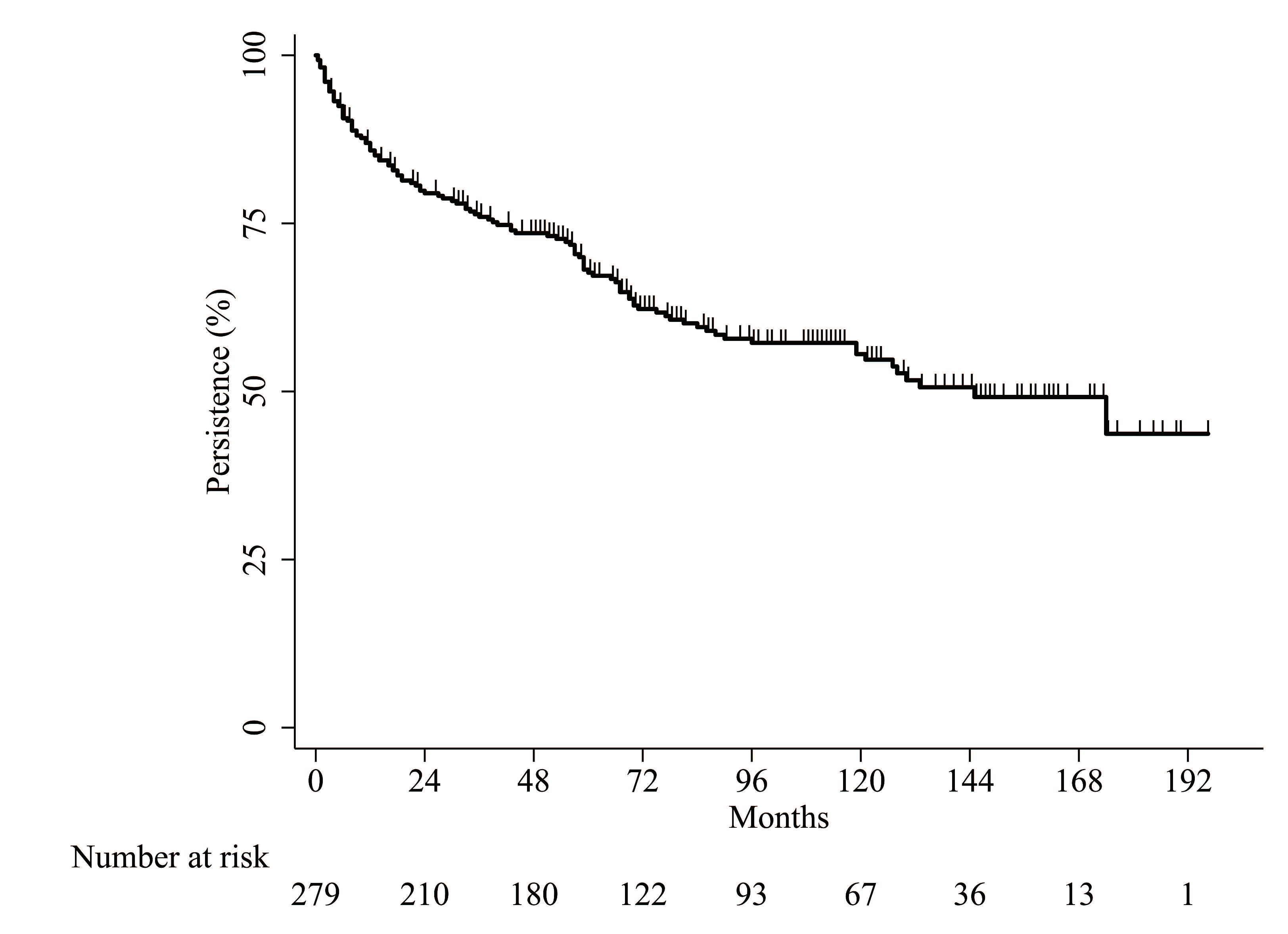

Supplement: Supplementary file 1 [file pharmaceuticals-16-00379-s001.zip › Supplmentary materials/Supplementary Figure 1.tif]
